# Gonadotropin-releasing hormone agonist ovulation trigger—beyond OHSS prevention

**DOI:** 10.1080/03009734.2020.1737599

**Published:** 2020-03-25

**Authors:** Juan Carlos Castillo, Thor Haahr, María Martínez-Moya, Peter Humaidan

**Affiliations:** a Department of Human Assisted Reproduction, Instituto Bernabeu, Alicante, Spain;; b Department of Clinical Medicine, Aarhus University, Aarhus, Denmark;; c The Fertility Clinic Skive, Skive Regional Hospital, Skive, Denmark

**Keywords:** Cancer, GnRH trigger, hCG, IVF, oocyte donation, ovulation trigger

## Abstract

In this review the advantages of the gonadotropin-releasing hormone agonist (GnRHa) trigger are discussed beyond those immediately associated with ovarian hyperstimulation syndrome (OHSS) prevention. The GnRHa trigger concept has sparked the development of novel protocols, enriching the assisted reproductive technology (ART) armamentarium for the benefit of present and future patients. Thus, GnRHa trigger already has a pivotal role, not only for the standard *in vitro* fertilisation (IVF) patient, but also for patient groups like oocyte donors, cancer patients, patients with poor ovarian reserve, and patients with immature oocyte syndrome and empty follicle syndrome. Herein, we discuss the importance of the GnRHa-elicited midcycle FSH surge and the potential improvement in oocyte yield and embryo competence.

## GnRHa trigger—improving oocyte yield and embryo competence?

In assisted reproductive technology (ART), hCG has been extensively used as a surrogate for the midcycle luteinizing hormone (LH). Due to its biochemical components and similar biological dynamics of LH, hCG binds to and activates the same receptor as LH, the LH/hCG receptor, thus ensuring excellent exposure of the follicle to LH activity. In contrast, when hCG is used for ovulation trigger there is a complete lack of the midcycle FSH surge and activity as seen during the natural midcycle surge of gonadotrophins (1). Until recently, clinicians have been relying solely on LH activity-dependent triggering of final oocyte maturation and, thus, have taken it for granted that the natural midcycle FSH surge was biologically redundant. However, since the early days of comparisons between gonadotropin-releasing hormone agonist (GnRHa) trigger and hCG trigger in *in vitro* fertilisation (IVF), studies reported the retrieval of more metaphase II (MII) oocytes and embryos after GnRHa trigger (2–4). Thus, Kol and Humaidan in 2010 questioned this paradigm, suggesting that the complex process of final follicular maturation and ovulation in IVF cycles might benefit from the synchronised interaction of both LH and FSH activity (5). Although the exact role of the FSH midcycle surge is not fully understood, it is known to: 1) promote nuclear oocyte maturation (6); 2) favour cumulus–oocyte communication, enhancing the network of gap junctions within the cumulus–oocyte complex (7); 3) stimulate cumulus expansion (8); and 4) favour the release of proteolytic enzymes involved in ovulation (plasmin) (9).

As observed previously, the surge of gonadotrophins elicited by a bolus of GnRHa differs from that of the natural midcycle of gonadotrophins in duration and profile. It has also been shown that the elicited flare of LH as well as FSH resembles the natural midcycle surge of gonadotrophins and was found to effectively stimulate final oocyte maturation and ovulation (10), leading to the development of competent embryos as recently demonstrated in PGT-A cycles (11). Moreover, studies in oocyte donors (12) and oncologic fertility-preservation patients (13,14) demonstrated a significant increase in MII oocytes and the number of good-quality embryos in GnRHa-triggered cycles as compared with hCG-triggered cycles. Nonetheless, it is worth noticing that others have not corroborated these findings (15–17).

Based on the previous observations, Lamb and co-workers explored the possible benefits of adding FSH to the hCG trigger bolus in a randomised placebo-controlled trial in a total of 188 IVF cycles (18). Thus, apart from the hCG trigger bolus, a total of 95 patients received 450 IU of FSH, whereas the remaining 93 patients received a placebo. There were higher retrieval rates (70% versus 57%) as well as fertilisation rates (63% versus 55%) in the FSH group of patients. Following the same line, Lin et al. (19) retrospectively analysed data from 376 normo-responder patients, undergoing GnRH-antagonist co-treatment, of whom a total of 191 received the so-called ‘dual trigger’ approach (20) compared with 187 patients receiving hCG only. More MII oocytes were retrieved within the dual trigger group (10.53 versus 8.03), and live-birth rates per embryo transfer were higher as well (41% versus 30%), suggesting a beneficial effect of adding FSH activity at the moment of final follicular maturation in IVF. More recently, the same group published a similar study (21), this time focussing on patients with diminished ovarian reserve (*n* = 427). The dual trigger group had higher oocyte fertilisation rates (73.1% versus 58.6%), clinical pregnancy rates (33.0% versus 20.7%), and live birth rates (26.9% versus 14.5%) when compared with the hCG-only trigger group. Of note, the pregnancy loss (17.4% versus 37.0%) and embryo transfer cancellation rate (6.1% versus 15.4%) were lower in the dual trigger group.

Taken together, the available data show how modifications in the trigger strategy based on physiology may have potential benefits for oocyte/embryo competence. At the time of trigger, it seems that FSH activity enhances a proper resumption of the meiotic processes of the oocyte, thus adding clinical value by improving oocyte recovery and fertilisation, and by improved pregnancy rates. Although the specific mechanisms need further exploration, these findings make the introduction of an FSH surge in addition to the surge of LH activity in IVF an attractive option for further improvement of success rates in IVF (5,18).

## Immature oocyte syndrome

In the human species, oocytes are created only during a small period of time during foetal life, after which they arrest in meiosis I (MI) until exposed to FSH and LH later in life. Generating embryos during IVF depends on multiple variables, and one of utmost importance is obtaining MII oocytes after the ovulation trigger. As noted previously, during ovarian stimulation (OS) for IVF, this process was traditionally induced by hCG which binds to the LH receptor acting as a surrogate for the natural midcycle LH surge. In this aspect, immature oocyte syndrome has been defined as a condition with more than 25% immature oocytes at retrieval after OS, despite the correct administration and timing of hCG (22). The aetiology of this obscure phenomenon is unknown, but has profound consequences for the couple, including a significant reduction in the chance of conceiving during IVF treatment (23).

Following a recent case report, describing a successful pregnancy by use of the dual trigger concept (20) in a patient with a history of immature oocyte syndrome (24), a retrospective study by Griffin et al. in 2012 reported the results of 27 women with a previous history of immature oocyte syndrome after hCG triggering (25). In the subsequent cycle, patients were triggered with a combination of GnRHa and hCG. By such means the number of MII oocytes increased compared with the previous cycle, resulting in the development of more transferable embryos. The odds of a mature oocyte retrieved for patients who received a dual trigger was 2.51 times higher, after controlling for confounding factors. The authors speculated that the retrieval of more MII oocytes might be associated with the presence of a surge of FSH/LH in addition to the hCG activity; yet the ongoing pregnancy rate per transfer was still disappointingly low (17.4%). In contrast, another small-size retrospective study of patients previously having a low proportion of MII oocytes (66%), despite normal response to OS, and who were subsequently submitted to a ‘double trigger’, reported not only a significantly higher number of mature oocytes (6.5 versus 3.6), but also an encouraging 50% clinical pregnancy rate (26). The ‘double trigger’ involves co-administration of GnRHa and hCG for final oocyte maturation, but at 40 and 34 h, prior to OPU, respectively (26).

Taken together, the presence of an FSH surge in addition to the LH and hCG surge seems to be a valuable tool in the armamentarium for the treatment of patients with immature oocyte syndrome. However, larger studies are needed to validate the reported retrospective results prior to its routine implementation.

## Empty follicle syndrome following hCG trigger

Empty follicle syndrome (EFS) was first described in 1986 in four patients in whom no oocytes were retrieved after apparently normal follicular development and appropriate oestradiol levels after OS (27). EFS is still a disturbing and challenging situation in IVF clinical practice, and the incidence varies among studies, ranging from 0.59% to 3.5% (28,29). Moreover, EFS seems to be associated with PCOS, GnRH-antagonist co-treatment (30), and diminished ovarian reserve (31). Interestingly, Revelli et al. suggested that EFS might not be a constant condition. Thus, in a study of 43 patients undergoing a second stimulation after EFS in the first cycle, a total of 37 patients (86%) obtained MII oocytes, although the stimulation protocols were similar, and a hCG trigger was used for the second cycle too (32). This could be caused by cycle-to-cycle variations in oocyte quality. However, a contribution from the statistical phenomenon ‘regression to the mean’ could also play a role as it is defined by the fact that an extreme variable in the first measurement is more likely to be closer to the mean in the next measurement.

In contrast, several case reports suggest a different picture. Thus, Lok et al. described a case presenting with two consecutive EFS (33). First, in a long GnRHa down-regulation protocol and subsequently in a GnRH antagonist protocol, both were triggered with 10,000 IU urinary hCG. In a second attempt, a single dose of GnRHa was used for final follicular maturation. Nine MII oocytes were retrieved from 10 follicles, and eight of these fertilised normally. Two good-quality embryos were used for fresh transfer, and four embryos were cryopreserved. A similar case has been described by Deepika et al. (34), with two consecutive EFS cycles with adequate follicular development and hormonal levels, and an uneventful oocyte pick-up after the use of an hCG trigger. In a third attempt, using GnRH antagonist co-treatment, the dual trigger option was chosen. A total of 10 MII oocytes were retrieved, and subsequently two-good quality blastocysts were transferred, leading to one successful live birth. However, the most puzzling case was described by Beck-Fruchter (35). Herein, the authors reported a case of a young woman with a normal karyotype and primary infertility of 25 months, submitted to IVF. After seven cycles including either a long GnRHa down-regulation protocol or GnRH antagonist co-treatment, resulting in normal follicular development and oestradiol levels, very unfavourable outcomes were obtained at retrieval, including four cycles with EFS and three cycles with 1–4 immature oocytes only. Of note, in these seven cycles rhCG (up to 13,000 IU) was used for trigger.

In the final successful cycle, a ‘double trigger’ (GnRHa, 40 h prior to OPU; and hCG, 34 h prior to OPU) was used, 16 MII oocytes were retrieved, and a total of 11 embryos developed; two embryos were transferred, and nine were cryopreserved; the fresh transfer resulted in the term birth of a healthy child. In the cases of Lok et al. and Beck-Fruchter et al. (33,35) it is difficult to clearly distinguish between the effect of the dual trigger concept versus an isolated action of the GnRHa bolus. Nevertheless, in view of the previous hCG failures, it seems reasonable to assume that some form of endogenous LH and/or the additive effect of the FSH surge could have played a role for the successful outcome.

All these observations suggest that EFS is a genuine entity. Albeit of obscure aetiology, EFS may represent a syndrome of impaired granulosa cell function, in which oocyte meiotic maturation is not resumed, cumulus expansion does not ensue, and the immature oocyte–cumulus complexes are resistant to follicular aspiration (35). However, evidence is mainly based on case reports and might suffer from publication bias. For the time being, it cannot be ruled out that in some patients the FSH surge is needed for optimal resumption of the oocyte meiotic processes, including EFS cases after hCG trigger. Finally, it is important to note that EFS can be encountered also after GnRHa trigger (28).

## Ovulation trigger in oocyte donation cycles

According to the European IVF-monitoring Consortium, oocyte donation cycles account for up to 32.4% of all ART treatments in some countries (36). The oocyte donation is usually performed in an altruistic manner by young and healthy women. Even though important complications account for less than 1% of all oocyte donation cycles (37), moderate to severe ovarian hyperstimulation syndrome (OHSS) might occur more frequently than anticipated, 0.87%−9.47% (37,38). As these patients do not proceed to embryo transfer, the incidence accounts exclusively for early-onset OHSS and are related to hCG trigger in the presence of a high ovarian response.

The use of GnRH antagonist co-treatment followed by GnRHa trigger has been shown to be the most advantageous protocol for the oocyte donor in terms of safety and efficacy. In oocyte donation cycles, outcomes after GnRHa trigger are similar to those of hCG trigger. In 2009, Galindo et al. reported similar oocyte maturation and fertilisation rates in 212 oocyte donors randomised to receive either GnRHa or rhCG for trigger (38). Furthermore, although donors at high risk of OHSS were excluded from randomisation, nine donors had mild OHSS and one donor severe OHSS in the rhCG group, whereas no OHSS cases were observed in the GnRHa trigger group. Additional randomised controlled trials have consistently reported similar outcomes. Importantly, the pregnancy rates in recipients are similar to those seen after hCG trigger (39,40). Further benefits for the oocyte donor population include: shorter duration of the luteal phase, reduced luteal phase discomfort and abdominal distension, and reduced ovary volume. All these added benefits contribute to a more friendly process for the oocyte donor (41–43), and GnRHa trigger should be the ‘gold standard’ for the oocyte donor.

## Ovarian torsion

Ovarian torsion (OT) happens when an ovary twists on its attachment to other structures. The development of an ovarian mass or ovarian enlargement is commonly related to the development of OT and may affect up to 7.5% of women who experience abdominal pain in emergency departments (44). OS may result in ovarian conditions that predispose patients to ovarian augmentation and torsion, with potentially significant consequences, as OT may lead to necrosis requiring ovariectomy, if left untreated.

In a recent retrospective cohort study (45), the incidence of OT and its subsequent complications of IVF cycles were explored. The analysis included more than 15,000 IVF cycles, using either hCG trigger and fresh embryo transfer or GnRHa trigger and elective frozen embryo transfer (eFET). As previously reported (46), OT was an infrequent complication (14 out of 15,577; 0.09%). It is worthy of note that of the 14 diagnosed OT cases, a total of 13 were diagnosed in the hCG-triggered fresh embryo transfer group and 1 in the GnRHa trigger eFET group (0.13% versus 0.018%, *p* < 0.049). Importantly, although the total oocyte number obtained in the GnRHa trigger group was higher than in the hCG trigger group, the incidence of OT was lower in the former. The authors correlated the lower OT rate to a lower OHSS rate in the GnRHa trigger group compared to the hCG trigger group (0.05% versus 2.4%, *p* < 0.001). In addition, others reported that the ovary will gain its normal volume faster and closer to the baseline prestimulation volume after GnRHa trigger (47,48), which may further contribute to a reduction in OT development.

## Fertility preservation in cancer patients

According to the Global Cancer Observatory (https://gco.iarc.fr/), breast cancer is the most common malignancy diagnosed in reproductive-age women worldwide, accounting for approximately 30% of all new cases reported in 2018. Since diagnostic tools and treatments have improved over the last decade, fertility preservation in this specific group of patients has gained importance. Considering the fact that a vast majority of breast cancers are hormone-dependent (49), attention has focussed on the supraphysiological oestradiol levels occurring during OS prior to oocyte preservation. Additionally, when hCG is used as a trigger agent, its luteotrophic effect will potentiate the function of multiple corpora lutea, further increasing oestrogen levels during the luteal phase.

In order to overcome these undesired effects of ovarian stimulation and ovulation trigger in breast cancer patients, OS protocols were developed involving the use of aromatase inhibitors (in addition to exogenous FSH) and GnRHa trigger. In their first small-size retrospective analysis Oktay et al. explored the use of either GnRHa trigger or hCG trigger in women with breast cancer who submitted to oocyte fertility preservation before chemotherapy (50). GnRHa trigger resulted in a considerable drop in luteal oestradiol levels compared with hCG trigger. Furthermore, more MII oocytes were retrieved, leading to the development of more 2PN embryos after GnRHa trigger. The same group in an extended analysis (13), including 129 breast cancer patients (46 in the GnRHa trigger group versus 83 in the hCG trigger group), confirmed their previous results in terms of more MII oocytes (77.3% versus 67.6%, *p* = 0.007) and a higher number of cryopreserved embryos (7.7 versus 5.4, *p* = 0.002) in the GnRHa trigger group. More recently, a larger retrospective cohort study (14) included 341 patient who underwent oocyte freezing for fertility preservation (75.3% breast cancer patients) and reported a higher number of MII oocytes and embryos cryopreserved (11.8 versus 9.9, *p* = 0.04; and 9.2 versus 6.4, *p* < 0.001) after GnRHa trigger.

Finally, the use of GnRHa trigger in the so-called ‘Random Start Controlled Ovarian Stimulation’ protocol was published, showing that GnRHa trigger effectively stimulates a flare of FHS/LH also in the luteal phase (51). The random start protocol has the potential to shorten the time to oocyte retrieval before oncology treatment.

In conclusion, on the basis of the reduced luteal oestrogen exposure after GnRHa trigger, the reduced risk of OHSS, and the improved cycle outcomes, the available evidence supports the use of GnRHa trigger in all women with breast cancer undergoing fertility preservation.

## Development of new protocols

In humans, the traditional model of follicular growth states that a single cohort of antral follicles develops during the follicular phase of a menstrual cycle. This classical theory of follicular recruitment has been challenged by Baerwald et al. by demonstrating the presence of two and even three wave-like changes in folliculogenesis during one single menstrual cycle, of which only one terminated in ovulation (52).

This new physiologic knowledge of folliculogenesis alongside previous experiences in the Random Start Controlled Ovarian Stimulation protocol paved the way for the development of the so-called ‘double stimulation’ protocol. A double stimulation protocol consists of two consecutive ovarian stimulations, the second one performed in the luteal phase, starting immediately after the first oocyte retrieval. This novel approach was initially used with promising results in a group of patients with poor ovarian reserve (53). From the double stimulation, a total of 26 women had 1–6 viable embryos cryopreserved, and 21 women underwent 23 cryopreserved embryo transfers, resulting in 13 clinical pregnancies. Importantly, in this novel protocol using GnRHa trigger in the follicular as well as the luteal phase of ovarian stimulation, it was found that the pituitary is able to respond adequately to a GnRHa trigger during the luteal phase, even in the presence of high circulating progesterone levels. Nonetheless, the FSH and LH surge induced by the same dose of GnRHa was higher after the first trigger compared with the second trigger. Other investigators, exploring the same concept of combined follicular and luteal phase OS, so-called DuoStim, reached the same conclusions (54). It was proposed that DuoStim would provide a better opportunity of retrieving oocytes in patients with poor ovarian reserve, in a shorter timespan as compared with conventional OS, and also suggested its potential use in oncologic patients in need of emergency fertility preservation. Finally, there is a recent publication reporting on the concept of double random ovarian stimulation, initiating OS regardless of the cycle day, and proceeding immediately with a second stimulation after the first retrieval in oncological patients (55).

## Reason for caution

A substantial part of the literature in the field of GnRHa trigger for the abovementioned patient sub-groups can be considered low-quality evidence. Hence, well conducted prospective trials are awaited in this area of IVF.

## Conclusions

GnRHa trigger has had a pivotal role in changing ovulation trigger policies worldwide, not only for the standard IVF patient, but also for patient groups like oocyte donors, cancer patients, patients with poor ovarian reserve, and patients with immature oocyte syndrome or empty follicle syndrome. Thus, GnRHa trigger plays an important role beyond OHSS prevention. Moreover, the GnRHa trigger concept has sparked the development of novel protocols, enriching the ART armamentarium for the benefit of present and future patients.
